# Pharmacokinetics, Safety and Tolerability of *Melissa officinalis *Extract which Contained Rosmarinic Acid in Healthy Individuals: A Randomized Controlled Trial

**DOI:** 10.1371/journal.pone.0126422

**Published:** 2015-05-15

**Authors:** Moeko Noguchi-Shinohara, Kenjiro Ono, Tsuyoshi Hamaguchi, Kazuo Iwasa, Toshitada Nagai, Shoko Kobayashi, Hiroyuki Nakamura, Masahito Yamada

**Affiliations:** 1 Department of Neurology and Neurobiology of Aging, Kanazawa University Graduate School of Medical Sciences, Kanazawa, Japan; 2 Department of Food and Life-Science, Takasaki University of Health and Welfare, Gunma, Japan; 3 Research Center for Food Safety, Graduate School of Agricultural and Life Sciences, The University of Tokyo, Tokyo, Japan; 4 Department of Environmental and Preventive Medicine, Kanazawa University Graduate School of Medical Sciences, Kanazawa, Japan; National Hospital of Utano, JAPAN

## Abstract

The aim of this study was to evaluate the safety, tolerability and pharmacokinetics of single dose of *Melissa officinalis* extract which contained rosmarinic acid, including food-effects in healthy individuals. A total of eleven healthy individuals were randomly assigned to treatment arms in the two studies [Study 1 (fasted state) and Study 2 (fed state)]. Rosmarinic acid in serum was measured by a coulometric detection method using High-Performance Liquid Chromatography electrochemical detector. The serum concentration of total rosmarinic acid peaked at 1 hour after administration of *Melissa officinalis *extract containing 500mg rosmarinic acid in fasted state, with a maximum serum concentration 162.20 nmol/ L. The area under the curve for intact rosmarinic acid was calculated from the serum concentration-time profile to be 832.13 nmol • hour/ L. Food intake increases area under the curve and delayed time at which the maximum serum concentration. Rosmarinic acid supplementation did not affect liver, kidney, or blood cell function parameters. No adverse event was reported by any of the participants due to the study treatment. Single dose of *Melissa officinalis *extract containing 500 mg rosmarinic acid appears to be safe and tolerable in healthy individuals. Food intake increased the exposure of rosmarinic acid and delayed absorption of rosmarinic acid in healthy individuals.

**Trial Registration:**

Trial Registration: UMIN-CTR UMIN000004997

## Introduction

An estimated 24 million people worldwide have dementia, the majority of whom are thought to have Alzheimer’s disease (AD) [1]. AD is characterized by extracellular parenchymal and vascular amyloid deposits comprising amyloid β-protein (Aβ) and the neurofibrillary tangle formed by microtubule-associated protein tau [2]. Continuing investigations of the pathogenic relationships of tau, Aβ, and AD suggest that oligomeric forms of Aβ play a seminal role in the disease causation [3, 4]. If so, the most efficacious therapeutic agents would target the aggregation or neurotoxic activity of these structures. This suggestion is consistent with the fact that current AD treatments, which block brain acetylcholine degradation or N-methyl-D-aspartate receptors, are not preventive or curative and produce only modest and temporary symptomatic effects [5].

Rosmarinic acid (RA) is an ester of caffeic acid and 3,4-dihydroxiphenyllactic acid. It has several interesting biological activities, e.g., antioxidant, anti-inflammatory, antimutagen, antibacterial, and antiviral [6]. For the anti-Aβ effect, there have been reports that RA reduces Aβ-induced neurotoxicity [7, 8]. We previously reported that RA dose-dependently inhibited Aβ fibrils formation from Aβ_40_ and Aβ_42_, as well as destabilizing preformed Aβ fibrils *in vitro* [9]. Recently, we showed that RA inhibited both Aβ_40_ and Aβ_42_ oligomerizations *in vitro* [10]. Moreover, our long term potentiation and long term depression assays using hippocampal slices indicated that RA decreased Aβ oligomer-induced synaptic toxicities [10] Long term potentiation and long term depression are considered important neurophysiological models of memory and learning and are used as experimental models of neuronal plasticity [11]. With anti-oligomeric effect in our previous *in vivo* study [12], RA was very promising because it inhibited both Aβ oligomerization and Aβ deposition.

RA is contained in various herbs such as perilla (*Perilla frutescens L*.) [13], rosemary (*Rosmarinus officinalis L*.) [14], sage (*Salvia officinalis L*.) [15], and lemon balm [*Melissa officinalis L*. *(M*. *officinalis)*] [16]. It was reported that extract of *M*. *officinalis* had good effect on cognitive performance in both of healthy participants and patients with AD with no side effects [17–19], though the amount of RA contained in the *M*. *officinalis* extract was not analyzed. Absorption and metabolism of single doses of *Perilla* extract containing 200mg RA were reported in healthy men [20]. Pharmacokinetics profiles containing food effects of *M*. *officinalis* extract including RA have not been reported.

The objective of this study was to evaluate the pharmacokinetics including food-effect and safety of *M*. *officinalis* extract containing 500mg, 250mg and 100mg of RA in healthy individuals.

## Methods

### Study participants

Healthy volunteers were recruited through flyers posted on Kanazawa University notice boards. Participants completed a detailed questionnaire that was developed to collect demographic, health, and dietary information.

Eligible participants in the study included both males and females who were aged above 20 years. Individuals were excluded if they had (i) a disease condition, such as dementia or liver, renal, or heart dysfunction; (ii) a history of cancer; (iii) allergies to polyphenols or any drug or food ingredient; or (iv) consumed any supplement containing rosmarinic acid within 15 days of the first administration of *M*. *officinalis* extract containing RA. Furthermore, women were excluded if they were pregnant or lactating. A total of eleven healthy individuals aged between 20 and 31 years were enrolled in the two studies [Study 1 (fasted state) and Study 2 (fed state)] using the same inclusion and exclusion criteria. The study statistician generated the random allocation sequence. All subjects were randomly allocated to treatment arms. Eight subjects were male (72.7%); three subjects were female. Seven subjects of the eleven completed both Study 1 and Study 2. Two subjects participated only in Study 1, and the another two subjects joined solely in Study 2. Therefore, nine subjects participated in Study 1, and nine in Study 2. Participants and investigators were masked to treatment allocation throughout the study.

The study was approved by the Kanazawa University Medical Ethics Review Board on February 16, 2011. Written informed consent was obtained from all study subjects upon enrollment. The authors confirm that all ongoing and related trials for this drug are registered. This study is registered at the University Hospital Medical Information Network Clinical Trials Registry (UMIN-CTR: UMIN000004997). The study protocol that was registered was exactly the same as the study protocol approved by the Kanazawa University Medical Ethics Review Board. Subjects were recruited starting in March 2011 and follow-up was completed in June 2011. This study was conducted in accordance to the Declaration of Helsinki in its currently applicable version. A safety monitoring team monitored the progress and data of the trial. The protocol for this trial and supporting CONSORT checklist are available as supporting information; see S1 CONSORT Checklist and S1 and S2 Protocols.

### Study design and intervention

This was a placebo-controlled trial to investigate the safety, tolerability and pharmacokinetics of *M*. *officinalis* extract containing RA. To date, the safety dosage of RA has not yet been investigated in humans. Acceptable daily intake (ADI) is a measure of the amount of a specific substance in food that can be orally ingested on daily basis over a lifetime without appreciable health risk [21]. An ADI value can be calculated from the no-observed-adverse-effect level (NOAEL) dividing by one hundred [21]. In our study using AD model transgenic mice (Tg2576), RA exhibited no adverse effect considered drug-related at a NOAEL of 1g/ kg BW/ day in the 10 months study [12]. When human body weight was assumed to be 50 kg, NOAEL was determined 50 g per day. Then, the ADI value of RA was calculated 500 mg per day.

The two studies were designed as follows.

**Fig 1:**
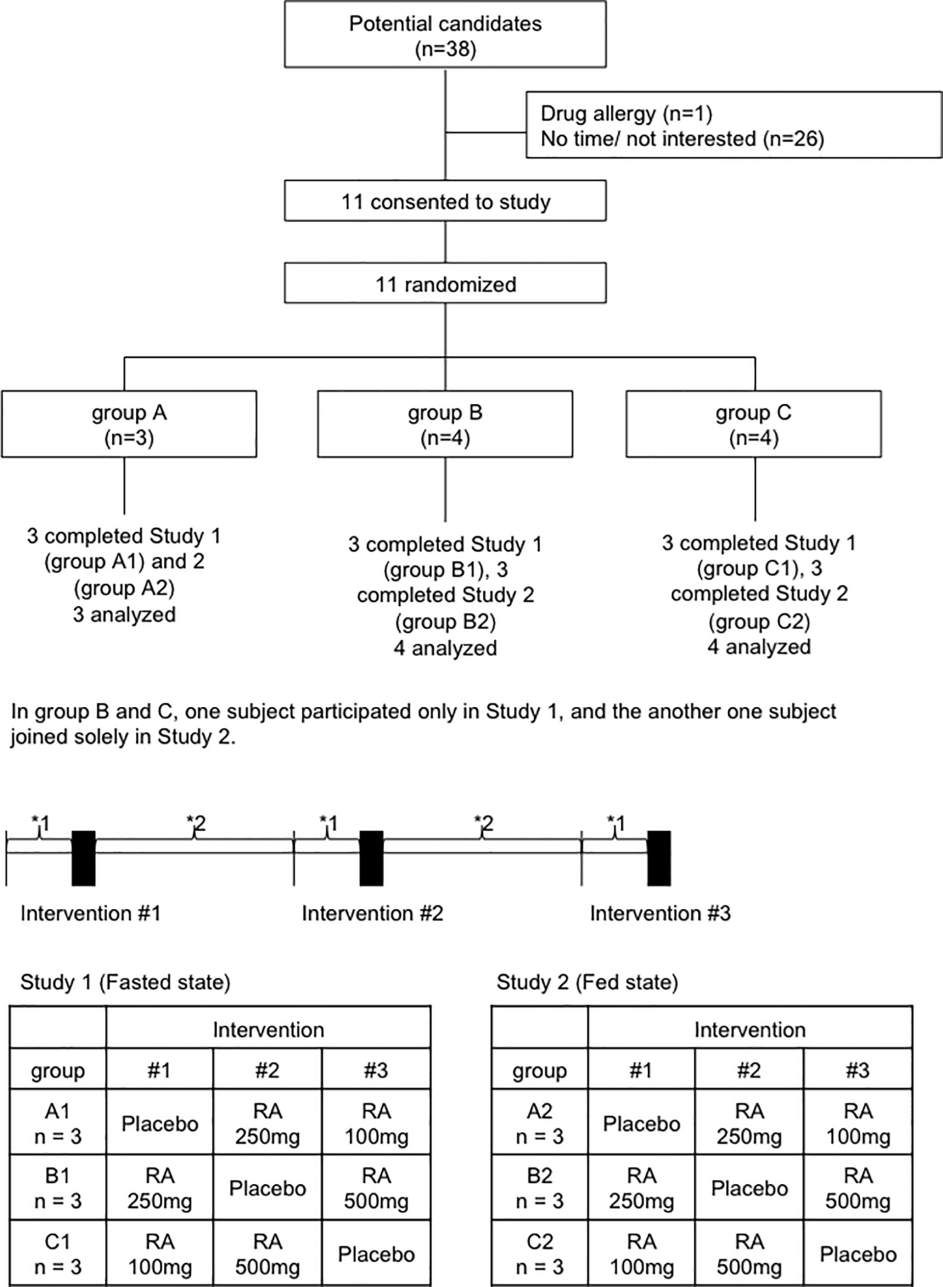
Experimental time course for each participant. Each participant completed three studies with a different RA dosage or placebo as designated by treatment group in the Latin square design. Blood samples were collected at baseline and 0.25, 0.5, 1, 2, 3, 6, 24, 48 hours after intake of RA or placebo on each intervention day. *1 indicates a 7-day controlled diet period. During this period, the participants were requested to consume foods that contain minimal amounts of polyphenols, such as rice and fish. * 2 indicates a 10-day wash-out period.

Study 1 (fasted state): Each participant received three separate interventions (investigation #1, #2, and #3) designed by groups A, B, or C in a 3 × 3 Latin square design with the order of consumption randomized and separated by a ten-day interval (wash-out period) (Fig 1). In details participants who allocated to group A were applied placebo in the investigation #1, and were applied *M*. *officinalis* extract containing RA 250 mg in the investigation #2 and were applied *M*. *officinalis* extract containing RA 100 mg in the investigation #3. Similarly, group B was applied *M*. *officinalis* extract containing RA 250 mg, placebo and *M*. *officinalis* extract containing RA 500 mg in the investigations #1, #2, and #3 respectively. Group C was applied *M*. *officinalis* extract containing RA 100 mg, *M*. *officinalis* extract containing RA 500 mg and placebo in these investigations #1, #2, and #3 in order given (Fig 1).

Each intervention was conducted for 10 days, consisting of a 7-day controlled diet period and a 3-day intervention period. In order to avoid the effects of polyphenols derived from other food sources during the intervention and controlled diet periods, participants were asked to consume foods that contain minimal amounts of polyphenols, such as rice and fish. The participants fasted for 12 h before the administration of *M*. *officinalis* extract containing RA or placebo.

Study 2 (fed state): In this fed state arm, placebo or *M*. *officinalis* extract containing RA was administered 30 min after completion of the standardized low-polyphenol meal, i.e. rice ball and clear soup. The other procedures of Study 2 were carried out in the same procedure as in the Study 1.

The primary outcome was to investigate pharmacokinetics of *M*. *officinalis* extract containing RA including food-effect in human. The secondary outcomes were to evaluate the safety and tolerability of *M*. *officinalis* extract containing RA.

Placebo and RA capsules (*M*. *officinalis* extract containing RA) were provided free from Maruzen Pharmaceuticals Co., Ltd., Japan. Lemon balm leaves (*M*. *officinalis*) were extracted with hot water and were purified with ethanol to obtain rosmarinic acid-rich extract. Each RA capsule contained 185 mg *M*. *officinalis* extract along with 50 mg RA, 60 mg lactose, and 5 mg calcium sterate. Each placebo capsule contained 210 mg lactose, 35 mg caramel, and 5 mg calcium stearate. There was no detectable RA content in the placebo capsule. Placebo capsules were made of the same size and color as the RA capsules. Because each RA capsule contained 50 mg RA, participants in the RA 100 mg, 250 mg, and 500 mg administration groups were allocated 2, 5, or 10 RA capsules, respectively. Participants in the placebo group were administered 2 placebo capsules.

### Measurements

For the purpose of the trial, physicians interviewed the participants, and performed clinical and physical examinations during their visits. They assessed the subjects’ physical condition and subjective symptoms and determined whether any adverse events were experienced. The health status of each subject was examined during physical examination. The examination parameters for the trial included height, weight, body mass index, and blood pressure, which were recorded from the subjects’ brachial region while they were seated.

Laboratory blood chemistry parameters, including a hematology (white blood cell count, red blood cell count, hemoglobin levels, and hematocrit and platelet count) and blood biochemistry [levels of aspirate aminotransferase (AST), alanine aminotransferase (ALT), lactate dehydrogenase (LDH), γ-glutamyltransferase (γ-GTP), total bilirubin (T-Bil), blood urea nitrogen (BUN), and creatinine (Cr)], were assessed with blood samples obtained at baseline and 48 h after the intake of *M*. *officinalis* extract containing RA or placebo. Baseline blood samples were collected from the intermediate cubital vein after the subjects had fasted for 12 h, followed by further samples being collected 0.25, 0.5, 1, 2, 3, 6, 24, 48 h after intake of *M*. *officinalis* extract containing RA or placebo. All the samples were processed and analyzed in a laboratory (SRL. Inc, Tokyo, Japan).

In the course of the clinical trial, adverse events associated with the study agents were self-reported by the participants, and blood analyses including blood cell counts and blood chemical parameters were monitored. All observed and self-reported adverse events, regardless of suspected causal relationship to the study treatments, were recorded on the adverse event form throughout the study.

Serum RA concentration is measured by High-Performance Liquid Chromatography-electrochemical detector analysis using an ESA coulometric detection system (ESA Inc., Boston, USA). Quantitative determination of RA is performed by using an external standard method [22], which verified that the detector response is linear for a concentration of up to 500 μmol/L for RA. In brief, 50 μL of serum were added 50 μL of 0.1 mol/L sodium acetate buffer (pH 5.0) and 200 μL of 0.83 mol/L acetic acid in methanol. The mixture was vortexed, sonicated, and centrifuged (at 8500g for 5 min at 4°C), and the supernatant was injected onto a High-Performance Liquid Chromatography-electrochemical detector C18 column (ODS150, MC Medical, Inc., Tokyo, Japan). The mobile phase A (solvent A) was 50 mmol/L sodium acetate containing 5% methanol (pH 3.0 adjusted with phosphoric acid), while the mobile phase B (solvent B) was 50 mmol/L sodium acetate containing 40% acetonitrile and 20% methanol (pH 3.5 adjusted with phosphoric acid). We used the following elution profile (0.6 mL/min): 0–28.5 min, linear gradient from 85% solvent A/15% solvent B to 20% solvent A/80% solvent B; 28.5–31 min, isocratic elution with 0% solvent A/100% solvent B; and 31–35 min, isocratic elution with 85% solvent A/15% solvent B. The eight electrode detector potentials were increased from 0 to 700 mV in increments of 100 mV. It was reported that the majority of RA were present in the blood as conjugated forms [20]. The difference in RA content before and after glucuronidase treatment was assumed to be attributed to the amount of the sulfate and glucuronide conjugates in the sample. Serum (50 μL) was mixed with 50 μL of glucuronidase type H-5 (Sigma-Aldrich. Inc. St. Louis. MO. USA) solution in 0.1 mol/L acetate buffer (pH 5.0) containing both 1.3 units of sulfatase and about 200 units of β-glucuronidase activity. The mixture was incubated at 37°C for 45 min.

### Statistical analyses

We chose the sample size to assess pharmacokinetics of *M*. *officinalis* extract containing RA in six subjects in each treatment arm. The one-way analysis of variance test was used to determine the significance of any differences among groups. The Chi-square test was used to determine the significance of gender. In the safety evaluation, defined as the blood analysis performed for each treatment arm, two-ways repeated measures analysis of variance was used to assess measurements. The SPSS software package (version 12.0J; SPSS Inc., Chicago, IL, USA) was used to perform statistical analyses. Area under the curve (AUC) for RA was calculated using the PK functions plug-in for Microsoft Excel [23]. The maximum serum concentration (C_max_), and time at which the maximum serum concentration was observed (T_max_) were determined from individual serum pharmacokinetic curves. We calculated AUC, C_max_, T_max_ for RA total, intact form and conjugate form, separately. The residual error was used to calculate the 90% confidence interval (CI) for the log-state fed-fasted difference. If the anti-logs of the estimate 90% CI of fed-fasted difference were within the limits of 0.80–1.25 and 0.70–1.43 for AUCs and C_max_, respectively, no food effect would be claimed [24]. Data were expressed as mean (SEM). A two-sided *p*-value of <0.05 was considered statistically significant in all the analyses.

## Results

### Safety and tolerability

Baseline characteristics were collected from all eleven eligible participants. With the exception of one, none of the participants withdrew before the end of the study; the withdrawing participant in the *M*. *officinalis* extract containing 100mg RA arm in fasted state dropped out halfway through the study because of illness induced by insufficient sleep. Nevertheless, the safety monitoring team speculated that this incident was unlikely to have been related to the study protocol. Because no adverse effects were recorded in the other intervention periods in which he took *M*. *officinalis* extract containing 500 mg RA and placebo in fasted state, and *M*. *officinalis* extract containing 100 mg, 500 mg RA and placebo in fed state. In the remaining participants, baseline characteristics were similar among all the different treatment groups (Table 1).

**Table 1:** Baseline demographic characteristics.

	Study 1 (Fasted state)					Study 2 (Fed state)				
Characteristics	group A1 (n = 3)	group B1 (n = 3)	group C1 (n = 3)	F value	*P* value	group A2 (n = 3)	group B2 (n = 3)	group C2 (n = 3)	F value	*P* value
Age (years)	25.00 (3.06)	21.67 (0.88)	22.67 (1.20)	F (2,6) = 0.760	0.508	25.00 (3.06)	24.33 (3.38)	22.67 (1.20)	F (2,6) = 0.195	0.828
Gender					0.325					0.526
Male, n	3	3	2			3	2	2		
Female, n	0	0	1			0	1	1		
Weight, kg	66.73 (2.16)	67.33 (4.56)	58.13 (8.21)	F (2,6) = 0.855	0.471	66.73 (2.16)	63.53 (7.45)	57.53 (8.68)	F (2,6) = 0.483	0.639
BMI, kg/m^2^	21.84 (0.82)	21.05 (1.68)	20.04 (1.34)	F (2,6) = 0.460	0.652	21.84 (0.82)	21.17 (1.64)	20.03 (1.35)	F (2,6) = 0.485	0.638
sBP, mmHg	126.67 (7.22)	128.67 (4.91)	117.67 (7.80)	F (2,6) = 0.752	0.511	126.67 (7.22)	121.67 (8.41)	117.33 (8.10)	F (2,6) = 0.347	0.720
dBP, mmHg	78.67 (5.78)	74.33 (3.67)	71.67 (2.33)	F (2,6) = 0.715	0.526	78.67 (5.78)	76.67 (5.24)	73.00 (1.53)	F (2,6) = 0.392	0.692

All values are the mean (SEM).

*P*-values of age, Weight, BMI, sBP and dBP were assessed among treatment arms (among groups A1, B1, and C1 for fasted state; among groups A2, B2, and C2 for fed state) using one-way repeated measures analysis of variance. *P*- values of Gender was assessed by chi-square test.

Abbreviation: BMI, body mass index. sBP, systolic blood pressure. dBP, diastolic blood pressure.

In the past history, one subject of group A had retinal detachment, one subject of group B mediastinal emphysema, another subject of group B dyslipidemia and renal calculus, and one subject of group C a history of surgery for endocardial deficiency. No subject had a habit of smoking. Two subjects of group B had a habit of drinking 1 or 2 bottles of beer per week. The other subjects had no habit of drinking.

Blood cell count data of one subject in group B were not available for coagulation. No significant changes were observed in the blood parameters between treatment arms (placebo, *M*. *officinalis* extract containing RA 100mg, 250mg or RA 500 mg) (Table 2).

**Table 2:** Effects of rosmarinic acid (*Melissa officinalis* extract) supplementation on blood cell counts and blood chemistry in Study 1 (fasted state).

	Placebo		RA 100 mg		RA 250 mg		RA 500 mg		F value	P value
	Baseline	48 hrs	Baseline	48 hrs	Baseline	48 hrs	Baseline	48 hrs		
**WBC (/μL)**	4825.00 (496.69)	4944.44 (481.06)	4540.00 (548.27)	4840.00 (828.61)	4683.33 (900.52)	4633.33 (631.75)	5400.00 (464.04)	4950.00 (381.88)	F (3,16) = 0.330	0.804
**RBC (×10** ^**4**^ **/μL)**	475.88 (7.95)	473.67 (12.28)	478.40 (7.52)	470.80 (7.04)	501.33 (16.15)	487.67 (13.54)	482.33 (13.65)	471.17 (17.64)	F (3,16) = 0.265	0.850
**Hb (g/dL)**	14.68 (0.36)	14.47 (0.38)	14.76 (0.40)	14.56 (0.45)	15.60 (0.47)	15.17 (0.28)	14.55 (0.39)	14.15 (0.54)	F (3,16) = 0.311	0.817
**Ht (%)**	43.45 (1.00)	42.74 (1.04)	43.74 (1.15)	43.00 (1.15)	45.58 (1.43)	44.08 (0.83)	43.00 (0.86)	41.70 (1.14)	F (3,16) = 0.317	0.813
**Plts (×10** ^**4**^ **/μL)**	24.38 (0.84)	25.58 (1.73)	24.16 (0.74)	23.60 (0.85)	22.93 (1.45)	24.05 (1.61)	24.38 (1.26)	23.83 (1.52)	F (3,16) = 0.736	0.546
**AST (U/L)**	17.56 (1.66)	19.00 (3.01)	17.40 (1.44)	17.80 (1.46)	22.33 (4.70)	21.33 (3.45)	18.00 (2.25)	18.00 (2.73)	F (3,17) = 0.751	0.537
**ALT (U/L)**	22.11 (5.97)	23.00 (7.92)	15.00 (2.92)	15.40 (3.04)	27.33 (11.44)	27.67 (11.31)	21.83 (6.73)	21.50 (7.20)	F (3,17) = 0.246	0.863
**LDH (U/L)**	152.22 (12.58)	151.56 (14.30)	156.00 (10.22)	151.60 (12.27)	158.33 (21.62)	152.50 (19.36)	146.00 (15.67)	138.33 (18.66)	F (3,17) = 0.288	0.833
**γ-GTP (U/L)**	19.33 (3.11)	17.67 (3.50)	13.00 (1.00)	12.00 (1.34)	22.83 (7.24)	21.17 (6.30)	21.00 (4.77)	19.17 (4.60)	F (3,17) = 0.070	0.975
**T-bil (mg/dL)**	0.81 (0.08)	0.73 (0.08)	0.64 (0.10)	0.54 (0.05)	0.80 (0.10)	0.67 (0.15)	0.67 (0.14)	0.60 (0.10)	F (3,17) = 0.348	0.791
**BUN (mg/dL)**	13.58 (1.19)	13.64 (1.16)	15.42 (1.95)	14.20 (1.40)	13.13 (1.17)	13.37 (1.50)	12.37 (1.17)	13.13 (0.92)	F (3,17) = 0.751	0.537
**Cr (mg/dL)**	0.83 (0.04)	0.80 (0.42)	0.80 (0.06)	0.81 (0.06)	0.82 (0.04)	0.84 (0.04)	0.80 (0.06)	0.77 (0.05)	F (3,17) = 1.983	0.155

All values are the mean (SEM).

*P*-values were assessed among treatment arms (Placebo, RA 100 mg, RA 250 mg, or RA 500 mg) using one-way analysis of variance.

Abbreviation: AST, aspartate aminotransferase levels; ALT, alanine aminotransferase levels; LDH, lactate dehydrogenase levels; γ-GTP, γ-glutamyltransferase levels; T-Bil, total bilirubin levels; BUN, blood urea nitrogen levels; Cr, creatinine levels; WBC, white blood cell count; RBC, red blood cell count; Hb, hemoglobin levels; Ht, hematocrit; Plt, platelet count.

The serum levels of AST, ALT, LDH, γ-GTP, and T-Bil (indicators of liver function) or those of BUN and Cr (indicators of kidney function) were not significantly affected by any of the interventions (*M*. *officinalis* extract containing 100 mg, 250 mg, 500 mg RA, or placebo) (Table 2).

### Pharmacokinetics of RA

**Fig 2:**
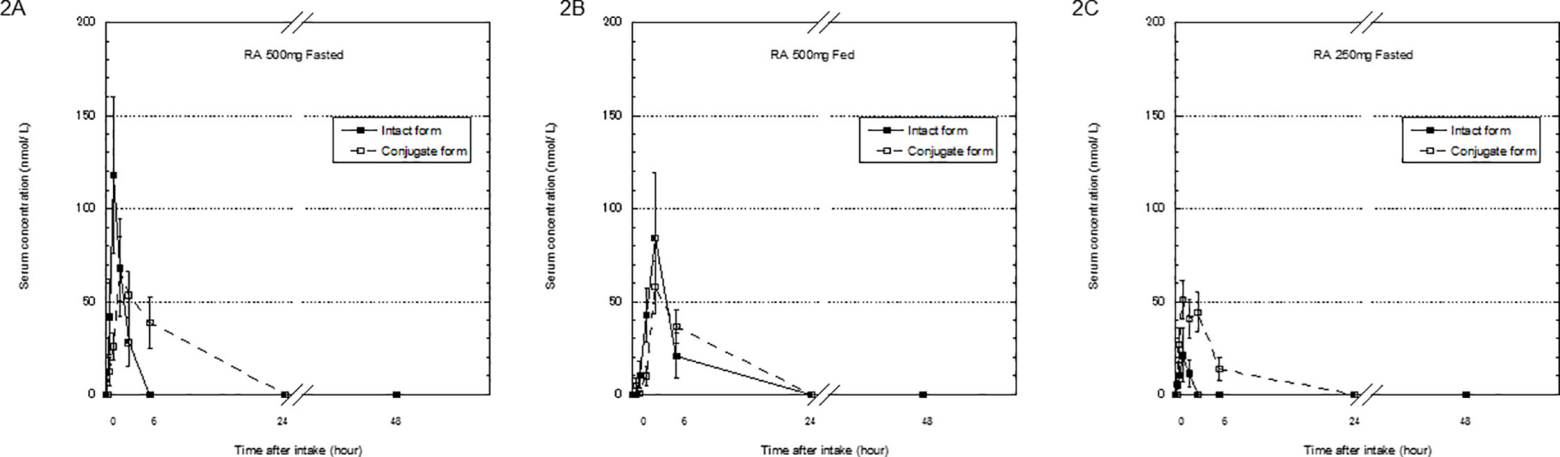
Serum concentration-time profiles in the serum concentration of rosmarinic acid (RA) before and 0.25, 0.5, 1, 2, 3, 6, 24 and 48 hours after intake of *Melissa officinalis* extract. Data show of intact and conjugated forms of RA after administration of 500mg RA [fasted (2A)/ fed state (2B)] and 250mg RA (fasted state) (2C). Each point is expressed as the mean ± SEM, n = 6.

In the placebo group, no RA was detected in the serum in all subjects neither in fasted nor fed state (data not shown). After administration of *M*. *officinalis* extract containing 100 mg RA, no RA was detected in serum samples from 4 of 6 subjects in fasted state or from 5 of 6 subjects in fed state (data not shown). The mean serum concentration-time profiles of RA after administration of a single dose of *M*. *officinalis* extract containing RA (500mg fasted/ fed state and 250mg fasted state) are shown in Fig 2.

Maximum concentration of total RA for the fasted regimens was reached 1h after oral administration of a single *M*. *officinalis* extract containing 250 mg RA and was 72.22 ± 12.01 nmol/ L (mean ± SEM), whereas no RA was detected in serum samples from 5 of 6 subjects in fed state. After administration of *M*. *officinalis* extract containing 500 mg of RA, total RA reached a maximum level of 162.20 ± 40.20 nmol/ L 1h and 142.20 ± 45.20 nmol/ L 3h, in fasted and fed state, respectively.

The noncompartmental pharmacokinetics analyses of intact and conjugated RA after administration of a single dose of *M*. *officinalis* extract containing RA (500 mg fasted/ fed state or 250 mg fasted state) are shown in Table 3.

**Table 3:** Rosmarinic acid (*Melissa officinalis* extract) pharmacokinetic parameters in serum under fasted and fed conditions.

	RA 250mg (fasted) n = 6	RA 500mg (fasted) n = 6	RA 500mg (fed) n = 6
AUC_0-48 Total_	354.88 (101.20)	832.13 (238.10)	945.03 (294.93)
AUC_0-48 Intact_	31.25 (17.72)	228.77 (82.65)	438.15 (206.09)
AUC_0-48 Conj_	323.63 (90.12)	603.36 (170.92)	513.88 (126.57)
C_max Total_	72.22 (12.01)	162.20 (40.20)	142.20 (45.20)
C_max Intact_	30.68 (12.88)	124.03 (39.13)	85.95 (34.97)
C_max Conj_	62.07 (8.55)	74.50 (15.93)	57.88 (14.32)
T_max Total_	1.00 (1.00, 1.00)	1.00 (1.00, 3.00)	3.00 (3.00, 3.00)
T_max Intact_	0.75 (0.00, 2.00)	1.00 (1.00, 3.00)	2.50 (0.00, 3.00)
T_max Conj_	1.00 (1.00, 3.00)	2.00 (2.00, 6.00)	3.00 (3.00, 3.00)

Values of C_max_ and AUC_0-48_ are the mean (SEM), n = 6. Values of T_max_ are median (range), n = 6.

Abbreviation: C_max_, maximum serum concentration; T_max_, time to reach the C_max_; AUC, area under the serum concentration-time curve.

When we administrated *M*. *officinalis* extract containing 500 mg RA in fasted state, the mean value of AUC_Total_ was increased to 2.0 times the AUC_Total_ of *M*. *officinalis* extract containing 250 mg RA administration in fasted state. Especially, the mean value of AUC_Intact_ of *M*. *officinalis* extract containing 500 mg RA fasted state was increased to 7.3 times the AUC_Intact_ of *M*. *officinalis* extract containing 250 mg RA fasted state. Median T_max Total_ values were similar in *M*. *officinalis* extract containing 250 mg and 500 mg RA fasted state. The mean value of C_max Total_ and C_max Intact_ of *M*. *officinalis* extract containing 500 mg RA fasted state were increased to 2.0 and 4.0 times the C_max Total_ and C_max Intact_ of *M*. *officinalis* extract containing 250 mg RA fasted state respectively (Table 3). Three subjects (group B) were included in both treatment arms [*M*. *officinalis* extract containing 250 mg RA (fasted) and 500 mg RA (fasted)], we showed the effect size along with its 95% CI (Table 4).

**Table 4:** Rosmarinic acid (*Melissa officinalis* extract) pharmacokinetic parameters in serum under fasted conditions in the same patients treated with RA 250 mg and RA 500 mg.

	RA 250mg (fasted) n = 3	RA 500mg (fasted) n = 3	F value	*P*-value	η^2^	95% CI
AUC_0-48 Total_	328.27 (127.84)	509.50 (323.03)	F (1,2) = 0.197	0.701	0.090	-1939.83 ~ 1577.36
AUC_0-48 Intact_	21.00 (10.50)	84.80 (27.73)	F (1,2) = 5.602	0.142	0.737	-179.76 ~ 52.17
AUC_0-48 Conj_	307.27 (123.23)	424.71 (296.02)	F (1,2) = 0.094	0.788	0.045	-1767.23 ~ 1532.34
C_max Total_	72.76 (11.78)	94.96 (40.07)	F (1,2) = 0.201	0.697	0.092	-234.99 ~ 190.59
C_max Intact_	24.51 (12.64)	58.78 (31.05)	F (1,2) = 1.785	0.313	0.472	-144.63 ~ 76.09
C_max Conj_	66.48 (15.21)	49.12 (20.25)	F (1,2) = 0.241	0.672	0.107	-134.88 ~ 169.61
T_max Total_	1.00 (1.00, 1.00)	1.00 (1.00, 3.00)	F (1,2) = 1.00	0.423	0.333	-3.54 ~ 2.20
T_max Intact_	1.50 (1.00, 2.00)	1.00 (1.0, 3.0)	F (1,1) = 1.00	0.500	0.500	-5.85 ~ 6.85
T_max Conj_	1.00 (1.00, 2.00)	3.0 (2.0, 6.0)	F (1,2) = 7.000	0.118	0.778	-6.13 ~ 1.46

Values of C_max_ and AUC_0-48_ are the mean (SEM), n = 3. Values of T_max_ are median (range), n = 3.

Abbreviation: C_max_, maximum serum concentration; T_max_, time to reach the C_max_; AUC, area under the serum concentration-time curve.

*P* values were assessed among treatment arms RA 250 mg (fasted) and RA 500 mg (fasted) using one-way repeated measures analysis of variance.

Regarding food-effect evaluation, significant increase in the mean value of AUC_Total_ (1.3 times) was observed with food intake. Statistical analysis result demonstrates that 90% CIs of AUC_Total_ ratio of *M*. *officinalis* extract containing 500 mg RA fed state to fasted state were significantly below the lower limit (0.80) and were significantly above the upper limit (1.25) (Table 5).

**Table 5:** Rosmarinic acid (*Melissa officinalis* extract) pharmacokinetic parameters and food-effect evaluation.

	PK parameters		Food-effect evaluation
	RA 500mg Fasted (n = 4)	RA 500mg Fed (n = 4)	Ratio (Fed/ Fasted), 90% CI
AUC_0-48 Total_	578.57 (268.49)	756.19 (197.26)	1.69, 0.63–4.52
C_max Total_	130.46 (49.47)	113.12 (37.65)	0.96, 0.35–2.65
T_max Total_	1.50 (1.00, 3.00)	3.00 (3.00, 3.00)	1.92, 1.01–3.63

Values of C_max_ and AUC_0-48_ are the mean (SEM). Values of T_max_ are median (range).

Abbreviation: C_max_, maximum serum concentration; T_max_, time to reach the C_max_; AUC, area under the serum concentration-time curve.

## Discussion

This is the first placebo-controlled randomized study to evaluate the safety, tolerability and pharmacokinetics of a single dose of *M*. *officinalis* extract including food-effects in healthy individuals. This study demonstrated that supplementation of a single dose of *M*. *officinalis* extract containing RA (100 mg, 250 mg, or 500 mg) did not raise any safety concerns with regards to clinical manifestations or routine blood tests.

Study 2 indicated that food intake affects C_max_, increases AUC and delayed T_max_. Food intake changes drug exposure through various ways, such as delaying gastric emptying, stimulating bile flow, changing the gastrointestinal pH value, increasing splanchnic blood flow, changing luminal metabolism, and physically or chemically interacting with the dosage form or the drug substance itself. Food intake would affect the absorption and increased the exposure of RA in healthy subjects. Food effect evaluation revealed 90% CI of fed-fasted difference were 0.63–4.52 and 0.35–2.65 for AUC_Total_ and C_max Total_, respectively. These results indicates food effects on pharmacokinetics exposure of RA have large differences among individuals. The sample size was small in the present study, further studies with large number subjects are required to reveal food effects on the pharmacokinetics of RA (*M*. *officinalis* extract). In our knowledge, the current study was the first evaluation of the pharmacokinetics of *M*. *officinalis* extract containing RA in a multiple-dose regimen with food in healthy volunteers.

Baba et al. reported the absorption and metabolic properties of single dose of *Perilla* extract containing 200 mg RA in healthy men [20]. No side effects or symptoms of toxicity have been reported with its use. The previous study reported that the concentration of intact and conjugated RA in the plasma were at most ~20 nmol/L and ~1200 nmol/L 0.5 h after administration of a single dose of 200 mg RA, respectively [20]. In contrast to our results, the majority of RA and its metabolites were present in the plasma as conjugated form [20]. In the present study, after a single 250 mg oral dose of RA, the C_max_ of conjugated RA was almost 2 times higher than that of intact RA, and the AUC of conjugated RA was approximately 10 times higher than that of intact RA. After oral administration of a single dose of 500mg RA in fasted state, the C_max_ of conjugated RA was similar to that of conjugated RA after a single dose of 250 mg RA intake. On the other hand, the C_max_ of intact RA was 1.7 times higher than that of conjugated RA after a single dose of *M*. *officinalis* extract containing 500 mg RA administration in fasted state. This findings suggest that conjugating high doses of RA takes time, and that the serum concentration of intact RA (non-conjugated RA) would be higher after oral administration of *M*. *officinalis* extract containing 500 mg RA than that of 250 mg.

Single doses of 300–1600 mg of *M*. *officinalis* extract have been reported to modulate mood and cognitive performance during acute administration in healthy young individuals using Cognitive Drug Research computerized test battery [17, 18]. In addition, it has been reported that *M*. *officinalis* extract, at a dose of 60 drops/day (500 μg citral/ml) for 16 weeks, produced a significantly better outcome on cognitive function in patients with mild to moderate AD [19]. However, these studies did not document the dosage of RA in *M*. *officinalis* treatments. The dried lemon balm leaves (*M*. *officinalis*) originally contained 4.1% RA [16]; on the basis of the finding from the report [16], one could assume that 300–1600 mg of *M*. *officinalis* might contain 12.3–65.6 mg RA. In the case of liquid infusion, 1 mg citral has been reported to contain 39.9 mg RA [16]; thus, 60 drops of *M*. *officinalis* presumably contains 120 mg RA. Since we did not include the assessment of mood and cognitive performance in the present study with healthy young individuals, further studies with cognitive assessments are required. Moreover, use of *M*. *officinalis* has not been associated with any side effects in previous studies. In the present study, the one adverse event observed in the *M*. *officinalis* extract containing 100 mg RA treatment arm was judged as unlikely to be related to the study protocol.

The present study was investigated in a young population, however, the final goal is to apply the *M*. *officinalis* in an aged population with age-related neurodegenerative disorders. There is the possibility that liver or renal dysfunction may influence the outcomes of metabolism of RA in older subjects. In addition, our previous work suggest that majority of RA is further metabolized and degraded into m-coumaric acid hydroxylated phenylpropionic acids by gut microbiota, which are then efficiently absorbed and distributed by the monocarboxylic acid transporter within the body [25]. It was reported that the composition of the microbiota is influenced more by environmental and social factors than the host’s genetic background [26]. We should consider that microbiota in older subjects might be different from that of younger ones, and the difference might influence the metabolism of RA.

In summary, we have demonstrated the safety, tolerability and pharmacokinetics of RA (*M*. *officinalis* extract). Our results reveal that a dose of *M*. *officinalis* extract containing 500 mg RA per day appears to be safe in humans. However, further studies are needed to investigate the safety of long-term RA use as well as its effects on cognitive performance in patients with AD.

## Supporting Information

S1 CONSORT Checklist CONSORT 2010 checklist. (DOC)

S1 Protocol Research protocol of pharmacokinetics of rosmarinic acid in healthy volunteers (English). (DOCX)

S2 Protocol Research protocol of pharmacokinetics of rosmarinic acid in healthy volunteers (Japanese). (PDF)

## References

[1] Ballard C, Gauthier S, Corbett A, Brayne C, Aarsland D, Jones E. Alzheimer’s disease. Lancet. 2011; 377: 1019–1031. 10.1016/S0140-6736(10)61349-9 21371747

[2] Hardy J, Selkoe DJ. The amyloid hypothesis of Alzheimer’s disease: Progress and problems on the road to therapeutics. Science. 2002; 297: 353–356. 12130773 10.1126/science.1072994

[3] Haass C, Selkoe DJ. Soluble protein oligomers in neurodegeneration: lessons from the Alzheimer’s amyloid β-peptide. Nat Rev Mol Cell Biol. 2007; 8: 101–112. 17245412 10.1038/nrm2101

[4] Ono K, Yamada M. Low-n oligomers as therapeutic targets of Alzheimer’s disease. J Neurochem. 2011; 117: 19–28. 10.1111/j.1471-4159.2011.07187.x 21244429

[5] Cummings JL. Alzheimer’s disease. N Engl J Med. 2004; 351: 56–67. 15229308 10.1056/NEJMra040223

[6] Petersen M, Simmonds MSJ. Rosmarinic acid. Phytochemistry 2003; 62: 121–125. 12482446 10.1016/s0031-9422(02)00513-7

[7] Alkam T, Nitta A, Mizoguchi H, Itoh A, Nabeshima T. A natural scavenger of peroxynitrites, rosmarinic acid, protects against impairment of memory induced by Aβ_25–35_ . Behav Brain Res. 2007; 180: 139–145. 17420060 10.1016/j.bbr.2007.03.001

[8] Iuvonoe T, Filippis DD, Esposito G, D’Amico A, Izzo AA. The spice sage and its active ingredient rosmarinic acid protect PC12 cells from amyloid-β peptide –induced neurotoxicity. J Pharmacol Exp Ther. 2006; 317: 1143–1149. 16495207 10.1124/jpet.105.099317

[9] Ono K, Hasegawa K, Naiki H, Yamada M. Curcumin has potent anti-amyloidogenic effects for Alzheimer’s β-amyloid fibrils in vitro. J Neurosci Res. 2004; 75: 742–750. 14994335 10.1002/jnr.20025

[10] Ono K, Li L, Takamura Y, Yoshiike Y, Zhu L, et al Phenolic compounds prevent amyloid β-protein oligomerization and synaptic dysfunction by site specific binding. J Biol Chem. 2012; 287: 14631–14643. 10.1074/jbc.M111.325456 22393064 PMC3340280

[11] Martin SJ, Grimwood PD, Morris GM. Synaptic plasticity and memory: an evaluation of the hypothesis. Annu Rev Neurosci. 2000; 23: 649–711. 10845078 10.1146/annurev.neuro.23.1.649

[12] Hamaguchi T, Ono K, Murase A, Yamada M. Phenoic compounds prevent Alzheimer’s pathology through different effects on the amyloid-β aggregation pathway. Am J Pathol. 2009; 175: 2557–2565. 10.2353/ajpath.2009.090417 19893028 PMC2789642

[13] Makino T, Ono T, Muso E, Honda G. Inhibitory effect of *Perilla frutescens* and its phenolic constituents on cultured murine mesangial cell proliferation. Planta Med. 1998; 64: 541–545. 9741301 10.1055/s-2006-957510

[14] al-Sereiti MR, Abu-Amer KM, Sen P. Pharmacology of rosemary (*Rosmarinus officinalis Linn*.) and its therapeutic potentials. Indian J Exp Biol. 1999; 37: 124–130. 10641130

[15] Areias F, Valentao P, Andrade PB, Ferreres F, Seabra RM. Flavonoids and phenolic acid of sage: influence of some agricultural factors. J Agric Food Chem. 2000; 48: 6081–6084. 11141272 10.1021/jf000440+

[16] Carnat AP, Carnat A, Fraisse D, Lamaison JL. The aromatic and polyphenolic composition of lemon balm (*Melissa officinalis L*. subsp. officinalis) tea. Pharm Acta Helv. 1998; 72: 301–305.

[17] Kennedy DO, Scholey AB, Tildesley NTJ, Perry EK, Wesnes KA. Modulation of mood and cognitive performance following acute administration of *Melissa officinalis* (lemon balm). Pharmacol Biochem Behav. 2002; 72: 953–964. 12062586 10.1016/s0091-3057(02)00777-3

[18] Kennedy DO, Wake G, Savelev S, Tildesley NTJ, Perry EK, Wesnes KA. Modulation of mood and cognitive performance following acute administration of single doses of *Melissa Officinalis* (Lemon Balm) with human CNS nicotinic and muscarinic receptor-binding properties. Neuropsychopharmacology. 2003; 28: 1871–1881. 12888775 10.1038/sj.npp.1300230

[19] Akhondzadeh S, Noroozian M, Mohammadi M, Ohadinia S, Jamshidi AH, Khani M. *Melissa officinalis* extract in the treatment of patients with mild to moderate Alzheimer’s disease: a double blind, randomized, placebo controlled trial. J Neurol Neurosurg Psychiatry. 2003; 74: 863–866. 12810768 10.1136/jnnp.74.7.863 PMC1738567

[20] Baba S, Osakabe N, Natsume M, Yasuda A, Muto Y, Hiyoshi K, et al Absorption, metabolism, degradation and urinary excretion of rosmarinic acid after intake of *Perilla frutescens* extract in humans. Eur J Nutr. 2005; 44: 1–9. 15309457 10.1007/s00394-004-0482-2

[21] Faustmen EM, Omenn GS. General principles of toxicology: Risk assessment In: Klaassen CD, editor. Casarett and Doull’s toxicology. McGraw-Hill Companies 1996, pp. 75–88.

[22] Konishi Y, Hitomi Y, Yoshida M, Yoshioka E. Pharmacokinetic study of caffeic and rosmarinic acids in rats after oral administration. J Agric Food Chem. 2005; 53: 4740–4746. 15941309 10.1021/jf0478307

[23] Usansky J, Desai A, Tang-Liu D. *PK functions for Microsoft Excel* [on line] 2011. Available: www.coomer.org/pkin/soft.html. Accessed 2014 May 22.

[24] Center for Drug Evaluation and Research (CDER), Food and Drug Administration (FDA) *Waiver of in vivo bioavailability and bioequivalence studies for immediate-release solid oral dosage forms based on a biopharmaceutics classification system* [on line] 2000. Available: www.fda.gov/. Accessed 2014 May 22.

[25] Konishi Y, Kobayashi S. Transepithelial transport of rosmarinic acid in intestinal caco-2 cell monolayers. Biosci Biotechnol Biochem. 2005; 69: 583–591. 15784988 10.1271/bbb.69.583

[26] Stahringer SS, Clemente JC, Corley R, Hewitt J, Knights D, Kranter KS. Nuture trumps nature in a longitudinal survey of salivary bacterial communities in twins from early adolescence to early adulthood. Genome Res. 2012; 22: 2146–2152. 10.1101/gr.140608.112 23064750 PMC3483544

