# Supplementary material for: Pharmacokinetics, Safety and Tolerability of Melissa officinalis Extract which Contained Rosmarinic Acid in Healthy Individuals: A Randomized Controlled Trial
Source: PLoS One. 2015 May 15;10(5):e0126422. doi: 10.1371/journal.pone.0126422 (PMC4433273; doi:10.1371/journal.pone.0126422)
Supplement: S1 Protocol — (DOCX) [file pone.0126422.s002.docx]

**RESEARCH PROTOCOL**

**Kanazawa University Medical Ethics Review Board**

**Approved on Feb 16, 2011**

Title: Pharmacokinetics of rosmarinic acid in healthy volunteers

Principal investigator Masahito Yamada (Department of Neurology, Neurobiology of Aging, Kanazawa University Graduate School of Medical Sciences)

Contributor

Kazuo Iwasa (Department of Neurology, Neurobiology of Aging, Kanazawa University Graduate School of Medical Sciences)

Kenjiro Ono (Department of Neurology, Neurobiology of Aging, Kanazawa University Graduate School of Medical Sciences)

Moeko Shinohara (Department of Neurology, Neurobiology of Aging, Kanazawa University Graduate School of Medical Sciences)

Shoko Kobayashi (Research Center for Food Safety, Graduate School of Agricultural and Life Sciences, The University of Tokyo)

Toshiomi Nagai (Department of Food and Life-Science, Takasaki University of Health and Welfare)

**1. Summary**

*Background*

An estimated 24 million people worldwide have dementia, the majority of whom are thought to have Alzheimer’s disease (AD). AD is characterized by extracellular parenchymal and vascular amyloid deposits comprising amyloid β protein (Aβ) and the neurofibrillary tangle formed by microtubule-associated protein tau.

Rosmarinic acid (RA) is an ester of caffeic acid and 3,4-dihydroxiphenyllactic acid. It has several interesting biological activities, e.g., antioxidant, anti-inflammatory, antimutagen, antibacterial, and antiviral [1]. We previously reported that RA dose-dependently inhibited Aβ fibrils formation from Aβ_40_ and Aβ_42_, as well as destabilizing preformed Aβ fibrils *in vitro* [2]. Recently, we showed that RA inhibited both Aβ_40_ and Aβ_42_ oligomerizations *in vitro* [3]. Pharmacokinetics profiles containing food effects of *M. officinalis* extract including RA have not been reported.

*Objectives*

The objective of this study was to evaluate the pharmacokinetics including food-effect and safety of *M. officinalis* extract containing 500mg, 250mg and 100mg of RA in healthy individuals.

*Methods*

Participants will be randomly assigned to treatment arms in the two studies [Study 1 (fasted state) and Study 2 (fed state)].

**2. Eligibility**

Healthy volunteers will be recruited through flyers posted on Kanazawa University notice boards. Participants will complete a detailed questionnaire that is developed to collect demographic, health, and dietary information. Eligible participants in the study included both males and females who are aged above 20 years. Individuals are excluded if they have (i) a disease condition, such as dementia or liver, renal, or heart dysfunction; (ii) a history of cancer; (iii) allergies to polyphenols or any drug or food ingredient; or (iv) consumed any supplement containing rosmarinic acid within 15 days of the first RA administration. Furthermore, women are excluded if they were pregnant or lactating.

**3. Target sample size**: We chose the sample size (n=9~18) to assess the primary objective of pharmacokinetics of RA.

**4. Methods**

This is a placebo-controlled trial to investigate the safety, tolerability and pharmacokinetics of RA.

**5. Study design**

5.1. Study 1 (fasted state)

Each participant will receive three separate interventions (investigation #1, #2, and #3) designed by groups A, B, or C in a 3 × 3 Latin square design with the order of consumption randomized and separated by a ten-day interval (wash-out period) (Figure 1). In details participants who allocated to group A are applied placebo in the investigation #1, and are applied RA 250 mg in the investigation #2 and are applied RA 100 mg in the investigation #3. Similarly, group B is applied RA 250 mg, placebo and RA 500 mg in the investigations #1, #2, and #3 respectively. Group C is applied RA 100 mg, RA 500 mg and placebo in these investigations #1, #2, and #3 in order given (Figure 1). Each intervention is conducted for 10 days, consisting of a 7-day controlled diet period and a 3-day intervention period. In order to avoid the effects of polyphenols derived from other food sources during the intervention and controlled diet periods, participants are asked to consume foods that contain minimal amounts of polyphenols, such as rice and fish. The participants will fast for 12 h before the administration of RA or placebo.

Figure 1.


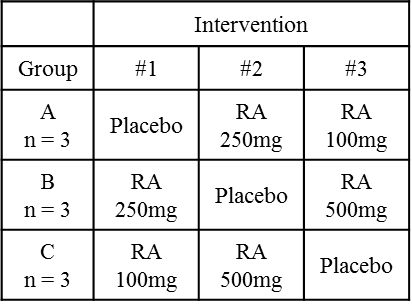


5.2. Study 2 (fed state)

In this fed state arm, placebo or RA will be administered 30 min after completion of the standardized low-polyphenol meal, i.e. rice ball and clear soup. The other procedures of Study 2 will be carried out in the same procedure as in the Study 1.

5.3. Polyphenol rich foods list

5.3.1. Rosmarinic acid rich foods

Lemon balm (*Melissa officinalis L.*), Rosemary (*Rosmarinus officinalis L.*), Perilla (*Perilla frutescens L.*), Sage (*Salvia officinalis L.*), Lavender (*Lavandula officinalis L.*), Tyme (*Thymus vulgaris L.*)

5.3.2 Polyphenol rich foods list (1)

Red wine, Beer, Green tea, Coffee, Cocoa, Chocolate, Black tea, Curry.

5.3.3. Polyphenol rich foods list (2)

Buckwheat, Soy, Eggplant, Onion, Broccoli, Grape, Strawberry, Apple, Raspberry, Olive, Sesame.

5.4 Placebo and RA capsules

5.4.1 Rosmarinic acid

Chemical name: Rosmarinic acid

Stractural diagram:


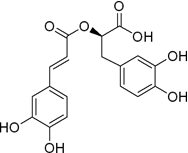


Molecular weight: 360.0845

Molecular formula: C_18_H_16_O_8_

RA capsules are provided free from Maruzen Pharmaceuticals Co., Ltd., Japan. Each RA capsule contained 185 mg *M. officinalis* extract along with 50 mg RA, 60 mg lactose, and 5 mg calcium sterate.

5.4.2 Placebo

Placebo capsules are provided free from Maruzen Pharmaceuticals Co., Ltd., Japan. Each placebo capsule contained 210 mg lactose, 35 mg caramel, and 5 mg calcium stearate. There is no detectable RA content in the placebo capsule.

5.5 Random allocation

The study statistician generate the random allocation sequence. All subjects will be randomly allocated to treatment arms.

5.6 Study methods

5.6.1 Study 1 (fasted state)

Group A: Placebo (intervention #1) - Rosmarinic acid 250 mg (intervention #2) – Rosmarinic acid 100 mg (intervention #3)

Group B: Rosmarinic acid 250 mg (intervention #1) - Placebo (intervention #2) – Rosmarinic acid 500 mg (intervention #3)

Group C: Rosmarinic acid 100 mg (intervention #1) - Rosmarinic acid 500 mg (intervention #2) – Placebo (intervention #3)

5.6.2 Study 2 (fed state)

Group D: Placebo (intervention #4) - Rosmarinic acid 250 mg (intervention #5) – Rosmarinic acid 100 mg (intervention #6)

Group E: Rosmarinic acid 250 mg (intervention #4) - Placebo (intervention #5) – Rosmarinic acid 500 mg (intervention #6)

Group F: Rosmarinic acid 100 mg (intervention #4) - Rosmarinic acid 500 mg (intervention #5) – Placebo (intervention #6)

5.7 Dosage of RA

To date, the safety dosage of RA has not yet been investigated in humans. It has been reported that M. officinalis extract, at a dose of 60 drops/day (500 μg citral /ml, presumably contains 240 mg RA) for 16 weeks, produced a significantly better outcome on cognitive function in patients with mild to moderate AD [4].

Acceptable daily intake (ADI) is a measure of the amount of a specific substance in food that can be orally ingested on daily basis over a lifetime without appreciable health risk [4]. An ADI value can be calculated from the no-observed-adverse-effect level (NOAEL) dividing by one hundred [5]. In our study using AD model transgenic mice (Tg2576), RA exhibited no adverse effect considered drug-related at a NOAEL of 1g/ kg BW/ day in the 10 months study [3]. When human body weight was assumed to be 50 kg, NOAEL was determined 50 g per day. Then, the ADI value of RA was calculated 500 mg per day.

5.7 Prohibited medications, supplements and other substances

The subjects has received any of the treatments about rosmarinic acid other than this trial or supplements contains polyphenol.

It was reported that lemon balm might induce depression in patient who undergo surgery by general anesthesia [6]. In case of general anesthesia, test schedule must be discontinued. It has been reported *M. officinalis* extract have sedative effects [7][8]. There is some possibility that *M officinalis* extract causes drowsiness. The combination use with sleep inducing drug or antianxiety drug and test capsule (rosmarinic acid or placebo) must be careful enough.

**6. The absorption and metabolic properties of Rosmarinic acid**

The previous study reported that the concentration of intact and conjugated RA in the plasma were at most ~20 nmol/L and ~1200 nmol/L 0.5 h after administration of a single dose of 200 mg RA, respectively [9].

**7. Test Schedule**

7.1.1 Physical condition and subjective symptoms

Physicians will interview the participants, and perform clinical and physical examinations during their visits. They will assess the subjects’ physical condition and subjective symptoms and determine whether any adverse events are experienced. The health status of each subject is examined during physical examination. The examination parameters for the trial included height, weight, body mass index, and blood pressure, which are recorded from the subjects’ brachial region while they are seated.

7.1.2 Blood tests

Laboratory blood chemistry parameters, including a hematology (white blood cell count, red blood cell count, hemoglobin levels, and hematocrit and platelet count) and blood biochemistry [levels of aspirate aminotransferase (AST), alanine aminotransferase (ALT), lactate dehydrogenase (LDH), γ-glutamyltransferase (γ-GTP), total bilirubin (T-Bil), blood urea nitrogen (BUN), and creatinine (Cr)], will be assessed with blood samples obtained at baseline and 48 h after the intake of RA or placebo. All the samples will be processed and analyzed in a laboratory (SRL. Inc, Tokyo, Japan). Baseline blood samples will be collected from the intermediate cubital vein after the subjects had fasted for 12 h, followed by further samples being collected 0.25, 0.5, 1, 2, 3, 6, 24, 48 h after intake of RA or placebo. Serum RA concentration is measured in Takasaki University of Health and Welfare (Gunma, Japan).

7.2 Adverse events

In the course of the clinical trial, adverse events associated with the study agents will be self-reported by the participants, and blood analyses including blood cell counts and blood chemical parameters are monitored. All observed and self-reported adverse events, regardless of suspected causal relationship to the study treatments, are recorded on the case report form (CRF) throughout the study.

7.3 Test schedule

7.3.1 Study 1 (fasted state)

7.3.2 Study 2 (fed state)

**8. Discontinued criteria**

A subject may withdraw (or be withdrawn) from the study prematurely for the following

reasons; (i) subject voluntarily discontinues participation in the study (consent withdrawal), (ii) unacceptable adverse events, (iii) other (must be specified).

**9. Adverse events**

9.1 Definition of Adverse Events (AE)

An AE is any untoward medical occurrence in a clinical investigation subject, temporally associated with the use of a medicinal product, whether or not considered related to the medicinal product.

9.2. Definition of Severe adverse events (SAE)

An SAE is any untoward medical occurrence that, at any dose: (i) Results in death, (ii) Is life-threatening, (iii) Requires hospitalization or prolongation of existing hospitalization, (iv) Results in disability/incapacity

9.3 Monitoring AEs

Subjects will be questioned and/or examined by the investigator for evidence of AEs. The questioning of subjects with regard to the possible occurrence of adverse events will be generalized such as, “How have you been feeling since your last visit?” The presence or absence of specific AEs should not be elicited from subjects.

9.4 Record of AEs

AEs, actions taken as a result of AEs, and follow-up results must be recorded in the CRF.

9.5 AEs after the clinical trial

If SAEs including death is any untoward medical occurrence in a clinical investigation subject after the clinical trial, the investigator will report the secretariat.

9.6 Expedited reporting

Any occurrence of SAEs in a subject in the trial must be reported expeditiously to the Kanazawa University Medical Ethics Review Board.

**10. Assessment**

10.1 Primary Endpoint

The primary outcome is to investigate pharmacokinetics of RA (M. officinalis extract) including food-effect in human.

10.2 Secondary Endpoint

The secondary outcomes are to evaluate the safety and tolerability of RA.

**11. Data analysis and statistical considerations**

11.1 Primary outcomes (Pharmacokinetics)

The maximum serum concentration (C_max_), and time at which the maximum serum concentration was observed (T_max_) will be determined from individual serum pharmacokinetic curves. AUC calculates it using trapezolital rule, expressed in mean± standard errors according to dose group (100 mg/ 250 mg/ 500 mg RA and placebo). We will compare Study 1 (fasted state) and Study 2 (fed state) of pharmacokinetics.

11.2 Secondary outcomes

In the safety evaluation, AEs compared between dose groups. Fisher’s exact test will be used to determine the significance of any differences among the 100 mg, 250 mg, 500 mg RA, and placebo groups.

11.3 Subject populations to be analyzed

The full analysis set population will serve as the primary population for the analysis of efficacy and safety data in this trial.

**12. CRF**

A CRF must be completed for all subjects who have given informed consent. All entries into CRFs are the responsibility of the investigator and must be completed by the investigator.

**13. Ethical conduct of the trial**

The trial must be conducted in accordance with Good Clinical Practice (GCP) and the Declaration of Helsinki.

13.1 Subject information and consent

The details of the protocol must be provided in written format and discussed with each potential subject, and written informed consent must be obtaining informed consent, the information must be provided in language and terms understandable to the subject.

13.2 Possible risks

Use of *M. officinalis* extract has not been associated with any side effects in previous studies [4]. It has been reported *M. officinalis* extract have sedative effects [7][8]. There is some possibility that *M officinalis* extract causes drowsiness.

13.3 Indemnification and reparation for health hazard

Each subject pays the medical expenses when healthy damage occurred.

**14. Expense**

There is not the funding from the company

**15. In case of changing the protocol**

In case of changing the protocol, the new protocol must be approved by the Kanazawa University Medical Ethics Review Board.

**16. Trial documents and records retention**

During the trial and after termination of the trial, the investigator must maintain all documents and records relating to the conduct of the trial. This documentation includes, but is not limited to, protocols, CRFs and laboratory data.

**17. Use of trial information in a publication**

The investigator has the right to publish results of the trial. Kanazawa university has intellectual property rights of this study.

**18. Research team**

Principal investigator Masahito Yamada (Department of Neurology, Neurobiology of Aging, Kanazawa University Graduate School of Medical Sciences)

Contributor Kazuo Iwasa, Kenjiro Ono, Moeko Shinohara, Shoko Kobayashi, Toshiomi Nagai

**19. References**

1) Petersen M, Simmonds MS: Rosmarinic acid. Phytochemistry 2003, 62: 121-125.

2) Ono K, Yoshiike Y, Takashima A, et al. Potent anti-amyloidogenic and fibril-destabilizing effects of polyphenols in vitro: implications for the prevention and therapeutics of Alzheimer’s disease. J Neurochem 2003, 87: 172-181.

3) Hamaguchi T, Ono K, Murase A, et al. Phenoic compounds prevent Alzheimer’s pathology through different effects on the amyloid-β aggregation pathway. Am J Pathol 2009, 175: 2557-2565.

4) Akhondzadeh S, Norozian M, Mohammadi M, et al. Melissa officinalis extract in the treatment of patients with mild to moderate Alzheimer’s disease: a double blind, randomized, placebo controlled trial. J Neurol Neurosurg Psychiatry 2003, 74: 863-866.

5) Merrill RA. Regulatory toxicology. In: Klaassen CD (ed) Toxicology, the basic science of poisons, 5th edn. McGraw-Hill, New-York, 1996, 1011-1023.

6) Natural medicines comprehensive database (available at: http://www.naturaldatabase.com/)

7) Kennedy DO, Scholey AB, Tildesley NT, et al. Modulation of mood and cognitive performamce following acute administration of Melissa officinalis (lemon balm). Pharmacol Biochem Behav 2002; 72: 953-964.

8) Kennedy DO, Wake G, Savelev S,et al. Modulation of mood and cognitive performance following acute administration of single doses of Melissa Officinalis (Lemon Balm) with human CNS Nicotinic and Muscarinic Receptor-Binding Properties. Neuropsychopharmacology 2003; 28: 1871-1881.

9) Baba S, Osakabe N, Natsume M, et al. Absorption, metabolism, degradation and urinary excretion of rosmarinic acid after intake of Perilla frutescens extract in humans. Eur J Nutr 2005, 44: 1-9.
