# Supplementary material for: Pharmacokinetics, Safety and Tolerability of Melissa officinalis Extract which Contained Rosmarinic Acid in Healthy Individuals: A Randomized Controlled Trial
Source: PLoS One. 2015 May 15;10(5):e0126422. doi: 10.1371/journal.pone.0126422 (PMC4433273; doi:10.1371/journal.pone.0126422)
Supplement: S2 Protocol — (PDF) [file pone.0126422.s003.pdf]

研究計画書

健常人における、ロスマリン酸の薬物動態に関する検討

2011/2/21 版

目次

1. 概要 2
2. 適格基準 3
3. 目標数と研究実施期間 3
4. 研究方法 3
5. 試験デザイン 4
6. ロスマリン酸の代謝・排泄 7
7. 試験スケジュール 7
8. 試験の中止基準 9
9. 有害事象の評価と報告 9
10. 評価項目 10
11. 統計的事項 11
12. 症例報告書の記入と報告 11
13. 倫理的配慮 12
14. 費用について 13
15. 実施計画の変更について 13
16. 記録の保存について 13
17. 研究成果の帰属と結果の公表 13
18. 研究組織 13
19. 参考文献 14

1. 概要

【背景】

超高齢化社会の到来が確実視されているわが国において、アルツハイマー病 (AD) に代表される認知症の予防、治療法の確立は非常に重要である。AD の主病態は大脳皮質のアミロイドβ蛋白 (Aβ) 沈着であり、Aβ凝集抑制効果をもつ化合物は AD 治療薬の候補となりうる。

ロスマリン酸は Melissa officinalis (レモンバーム)などハーブ類に多く含まれる植物性ポリフェノールであり、抗酸化、抗炎症、抗細菌、抗ウイルス活性を有するとされている [1]。山田らは試験管内のアミロイド線維形成反応モデルを用いて、ポリフェノール類が Aβ線維形成、伸長を抑制することおよびすでに形成された Aβ線維を不安定化することを明らかにした [2]。さらに AD モデル動物（Tg2576 マウス）にロスマリン酸などのポリフェノールを投与したところ、ロスマリン酸で脳内 Aβ沈着の有意な減少、Aβオリゴマーの有意な減少を認め、ロスマリン酸に AD 病理の予防効果があることを証明した [3]。

このようにロスマリン酸は AD 治療効果が期待され、副作用はすくないとおもわれるが、AD 患者は身体予備能の低下した高齢者が多く AD 予防および治療効果を得るには長期間の服用が必要になることから、安全性および忍容性の確認が必要である。

そこで、軽症～中等症の AD 患者にロスマリン酸（200mg から漸増し 500mg/日）を投与し、ロスマリン酸経口摂取の安全性および忍容性、代謝動態と認知機能の変化を評価するため、「アルツハイマー病患者における、ロスマリン酸の安全性と有効性に関する検討：二重盲検プラセボ試験」を計画し、金沢大学医学倫理員会にて既に承認を得ている（受付番号 932:平成 22 年 9 月 15 日承認）。

上記研究に使用予定のレモンバーム抽出ロスマリン酸製剤の健常人における薬物動態は明らかになっていない。

【目的】
本研究は健常人における、レモンバーム抽出ロスマリン酸製剤の薬物動態を明らかにすることを目的とする。

【方法】
金沢大学神経内科にて、健常人ボランティア約 9～18 名に対してロスマリン酸（125mg, 250mg または 500mg）を 1 回投与したときの薬物動態を比較検討する、無作為化二重盲検、プラセボ対照クロスオーバー試験である。試験は絶食下と食事摂取下で行われる。できる限り両方の試験に参加できる健常人ボランティアを募集する。

2. 適格基準

「選択基準」に該当し、「除外基準」に抵触しない症例で、本研究参加に自発的に文書にて同意した健常人ボランティアとする。

2.1 選択基準

(1). 同意日の年齢が 20 歳以上で性別は問わない。
(2). ロスマリン酸投与開始前に本人の文書同意が得られるもの。

2.2. 除外基準

下記のいずれかの項目に該当する患者は対象から除外する。
(1). ポリフェノール類を含有する栄養補助食品をロスマリン酸投与開始前 15 日以内に服用した者。
(2). AD を含む認知症性疾患を有している者。
(3). 重篤な心・肝・腎疾患を有している者。
(4). 悪性腫瘍の治療中の者。
(5). アルコールおよび薬物乱用の既往歴のある者。
(6). ポリフェノール類に薬物過敏症の既往のある者。
(7). 薬物および食物アレルギーの既往のある者。
(8). 他の治験に参加中の者。
(9). その他、研究責任医師または研究担当医師が不適当と認めた患者。

2.3. 選定方法

金沢大学宝町キャンパス内および金沢大学神経内科ホームページ上に臨床研究参加募集の掲示を行う。

3. 目標数と研究実施期間

健常人ボランティア　9～18 名以上
承認日～2015 年 3 月 31 日

4. 研究方法

本研究は、健常人ボランティアに対してロスマリン酸（125mg, 250ｍｇあるいは 500mg）を単回投与したときの薬物動態を比較検討する、無作為化二重盲検、プラセボ対照クロスオーバー試験である。

5. 試験デザイン

5.1. 絶食下における薬物動態の検討

本研究は 3 回の試験食品投与および血中ロスマリン酸濃度測定（study 1, 2, 3）からなる。健常人ボランティアは A,B,C 群のいずれかに無作為に割り付けられる。被験者は試験開始前 7 日間、ロスマリン酸を高濃度に含む食品の摂取を行わない。参加者は試験食品摂取前 12 時間から試験食品摂取後 3 時間までは絶食とする。また、試験食品摂取前 24 時間から摂取後 48 時間までポリフェノールを高濃度に含む食品（5.1.3. フェノール化合物を多く含む食品リスト参照）を避けて食事摂取をする。各 study では試験食品の投与と経時的な血中濃度採血を実施する。各 study の実施はそれぞれ 10 日以上あけることとする。

	study 1	Interbal 10days	study 2	Interbal 10days	study 3
GroupA (n=3)	Placebo		250mg		100mg
GroupB (n=3)	250mg		Placebo		500mg
Group C (n=3)	100mg		500mg		Placebo

5.2.食事摂取下における薬物動態の検討

本研究は 3 回の試験食品投与および血中ロスマリン酸濃度測定（study 4, 5, 6）からなる。健常人ボランティアは D,E,F 群のいずれかに無作為に割り付けられる。被験者は試験食品摂取前 7 日間、ロスマリン酸を高濃度に含む食品の摂取を行わない。参加者は試験食品摂取 30 分～15 分前に同じ食事（フェノール化合物を高濃度に含む食品を避けた食事）を摂取する。また、試験食品摂取前 24 時間から摂取後 48 時間までポリフェノールを高濃度に含む食品を避けた食事を摂取する（5.3. フェノール化合物を多く含む食品リスト参照）。各 study では試験食品の投与と経時的な血中濃度採血を実施する。各 study の実施はそれぞれ 10 日以上あけることとする。study 1- 3 と同じ被験者で study 4-6 を行う場合、study 3 と study 4 の間は 10 日以上あけることとする。

	study 4	Interbal 10days	study 5	Interbal 10days	study 6
Group D (n=3)	Placebo		250mg		100mg
Group E (n=3)	250mg		Placebo		500mg
Group F (n=3)	100mg		500mg		Placebo

5.3.フェノール化合物を多く含む食品リスト

5.3.1. 試験食品摂取 7 日前から試験食品摂取 48 時間後まで摂取をさける食品（飲料を含む）
ロスマリン酸を多く含むもの
　レモンバーム、ローズマリー、ラベンダー、タイム、セージ~~などのハーブ~~類、シソ、梅干

5.3.2.試験食品摂取 24 時間前から試験食品摂取 48 時間後まで絶対に摂取をさける食品（飲料を含む）

赤ワイン、ビールなどアルコール飲料全般、緑茶など日本茶全般、コーヒー、ココア、チョコレート、紅茶、カレー粉を使用した食品（カレーライスなど）

5.3.3.試験食品摂取 24 時間前から試験食品摂取 48 時間後までできる限り摂取をさける（少量の摂取は可能な）食品（飲料を含む）

穀類：そば

イモ類：ムラサキイモ、ベニイモ

豆類：大豆、黒大豆（黒豆）

野菜類：ナス、玉ねぎ、ブロッコリー、モロヘイヤ、朝鮮人参、ショウガ

果物類：ブルーベリー、プルーン、ブドウ、イチゴ、リンゴ、ラズベリー、コケモモ、クランベリー、オリーブ、柑橘類全般

調味料：ゴマ

5.4. 試験食品の概要
5.4.1. 被験食品（ロスマリン酸）について
　ロスマリン酸含有レモンバーム抽出物を用いる。レモンバームより抽出したロスマリン酸の含量を規格化し被験食品とする。

・化学名：rosmarinic acid

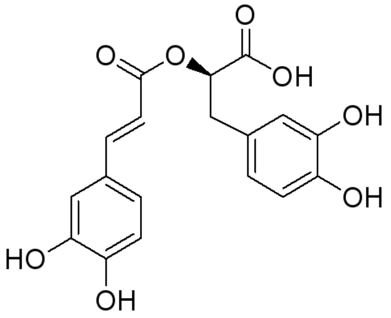

・構造式：
・分子量：360.0845
・分子式：$C_{18}H_{16}O_8$
・性状：カプセル
・含有量：1 カプセル中にロスマリン酸 50mg（乳糖，フレーバー，セルロースパウダー 50mg)
・ラベル：ロスマリン酸（試験研究用途・非売品）
・使用期限：2 年
・ロット番号：別途指定

・取り扱い上の注意：室温保存
・試験食品（レモンバーム抽出ロスマリン酸カプセル）は丸善製薬株式会社より無償提供を受ける。

5.4.2. プラセボ

1カプセル中乳糖、フレーバー、セルロースパウダーを配合したプラセボを使用する。二重盲検比較試験法にて実施するため、ロスマリン酸試験食品およびプラセボとも識別不能なカプセルを使用する。

プラセボカプセルについては丸善製薬株式会社より無償提供を受ける。

5.4. 3. 表示

試験食品の外箱には研究用である旨、試験食品数量、試験食品割付記号、保存方法、使用期限、注意事項および研究者名、住所、電話番号を表示する。

5.5. 無作為割付けについて

被験者は二重盲検下でA, B, C群あるいはD, E, F群のいずれかに割りつけられる。層別化因子は年齢、性別の2因子を考慮して割り付ける。

5.6. 試験方法

5.6.1. 絶食下における薬物動態の検討、投与群

(1). A群:プラセボ(study 1)-ロスマリン酸250mg(study 2)－ロスマリン酸 100mg(study 3)
3例

(2). B群:ロスマリン酸250mg(study 1)-プラセボ (study 2)-ロスマリン酸 500mg(study 3)
3例

(3). C群:ロスマリン酸 100mg(study 1)-ロスマリン酸500mg(study 2)－プラセボ(study 3)
3例

5.7.2. 食事摂取下における薬物動態の検討

(1). D群:プラセボ (study 4)-ロスマリン酸 250mg (study 5)-ロスマリン酸 100mg(study 6)
3例

(2). E群:ロスマリン酸250mg(study 4)-プラセボ (study 5)-ロスマリン酸 500mg(study 6)
3例

(3). F群:ロスマリン酸 100mg(study 4)-ロスマリン酸500mg(study 5)－プラセボ(study 6)
3例

5.6.2. 試験期間

5.6.2.1. 絶食下における薬物動態の検討

本研究の各 study (1, 2, 3) は試験食品投与24時間前から、投与後48時間の3日間行われ、study 1, 2, 3合わせて9日間からなる。各studyは10日以上間隔を空けて実施する。

5.6.2.2. 食事摂取下における薬物動態の検討

本研究の各 study (4, 5, 6) は試験食品投与24時間前から、投与後48時間の3日間行われ、study 4, 5, 6合わせて9日間からなる。各studyは10日以上間隔を空けて実施する。

［投与量の設定根拠］

1 日摂取量は海外の臨床試験で Melissa officinalis（レモンバーム)から抽出したロスマリン酸（約 240mg/日）を 21 人の軽度～中等度 AD 患者に 16 週間投与したところプラセボ群に比べ有意に認知機能改善をみとめ、プラセボ群に比して有意な有害事象はなかったことが報告されている[4]ことから、200mg/日から開始することにした。

また、Tg2576 マウス(メス)に 1g/kg・BW/日のロスマリン酸を 270 日経口投与した動物実験[3]では明らかな毒性は認められていない。食品においては、生涯にわたり毎日摂取し続けても影響が出ないと考えられる量を一日摂取許容量（acceptable daily intake, ADI）と定義し、動物実験で悪影響がみられなかった最大無毒性量（no observed adverse effect level, NOAEL）に安全係数*を乗じてもとめる。[5]今回 NOAEL を 1g/kg・BW/日とすると ADI は最大無毒性量×1/100(安全係数)であるから、10mg/kg・BW/日と計算される。したがってロスマリン酸 500mg/日の投与は安全と考えられる（平均的な成人の体重を 50ｋｇと仮定する）。
*安全係数には実験動物とヒトとの種の違い（10 倍）と個人差（10 倍）を考慮し、それらを乗じた 100 が用いられる。

5.7. 併用薬および併用療法

5.7.1. 併用禁止薬および禁止療法

・本研究で処方される以外のロスマリン酸、あるいはポリフェノール類を含有する栄養補助食品などを併用しない。
・全身麻酔を必要とする外科手術の際はレモンバーム摂取によりうつ状態が生じるおそれがあるといわれており[6]、臨床試験中に全身麻酔下の外科手術が必要になった場合には試験食品の投与を中断する。

5.7.2. 併用注意薬

・本研究で用いるロスマリン酸の原料となるレモンバームは弱い鎮静作用を有することが知られているが[7]、どの成分が鎮静作用をもつのかは明らかとなっていない[8]。本研究で用いるレモンバーム抽出ロスマリン酸においても、鎮静作用のある薬品やアルコールとの併用により、鎮静効果が高まる恐れがある[7]。そこで、睡眠導入剤や催眠鎮静薬、抗不安薬との併用する際は過鎮静に十分注意し、アルコールと共に摂取することは避けるよう指導する。

5.7.3. 併用可能薬・可能療法

(1). 研究担当医師により治療に必要と考えられる薬剤で、除外基準（1）に抵触しないもの。
(2). 研究期間中を通じて併用薬の種類、栄養補助食品などの種類、用法・用量は原則として変更しないこととする。

6. ロスマリン酸の代謝・排泄

Perilla frutescens（シソ）から抽出されたロスマリン酸のヒトにおける代謝・排泄に関しては、200mg 服用後 0.5 時間で血中濃度はピークに達し、methylated rosmarinic acid (methyl-RA), cafferic acid (CAA), ferulic acid (FA), m-coumaric acid (COA)に代謝され、6 時間以内に 75%が尿中へ排泄された[9]。

7. 試験スケジュール

7.1. 観察・検査・調査項目・実施期間

7.1.1. 被験者背景(スクリーニング時)

研究開始前の被験者背景を記録する。
観察項目：被験者名、性別、生年月日、身長、体重、BMI、バイタルサイン（血圧・脈拍・体温）、被験者の嗜好（喫煙、飲酒など）、既往歴（高脂血症、高血圧、糖尿病などの有無、治療歴）、薬物療法、薬剤の長期使用（処方薬および植物製剤や健康食品を含む市販されている医薬品）、薬剤に対するアレルギーまたは特異反応の既往を含む。

7.1.2. 血液検査

試験期間中の採血は、スクリーニング時、各 Study (Study 1-6)のベースライン、試験食品摂取 0.5 時間後、1 時間後、2 時間後、3 時間後、6 時間後、24 時間後、48 時間後 (各 Study で 8 回) おこなう。

7.1.2. 1. 血液検査(ロスマリン酸およびその代謝産物の濃度)

・血中ロスマリン酸およびその代謝産物の測定

ベースライン、試験食品摂取 0.5 時間後、1 時間後、2 時間後、3 時間後、6 時間後、24 時間後、48 時間後に採血をおこなう。ベースラインは試験食品投与前 1 時間以内とし、絶食下における薬物動態検討試験の際はベースラインの採血前 12 時間は絶飲食とする。測定は、高崎健康福祉大学で実施する。

7.1.2.2. 一般血液検査(スクリーニング時、試験食品摂取 1 時間前、48 時間後)

血液学的検査、血液生化学的検査を測定する。検査結果については臨床的意義のある異常変動か否かの判定とその理由およびロスマリン酸との因果関係の判定とその理由を記載する。

測定項目：

①血液学的検査：白血球数、赤血球数、ヘモグロビン、ヘマトクリット、血小板数

②血液生化学的検査：総蛋白、アルブミン、総ビリルビン、AST、ALT、γ-GTP、LDH、ALP、CPK、Na、K、Ca、クレアチニン、尿酸、血糖、LDL コレステロール、HDL コレステロール、トリグリセリド

測定は、エスアールエル (株) で実施する。

7.1.3. 食事、飲料

・食事

被験者は試験開始前 7 日間、ロスマリン酸を高濃度に含む食品の摂取を行わない。

被験者には試験食品摂取前 48 時間から摂取後 48 時間までに摂取したすべての食品、飲料、嗜好品について記載してもらう。

試験食品摂取 24 時間前から試験食品摂取 48 時間後まで、被験者はポリフェノールを高濃度に含む食品をさけて食事を摂取する。絶食下における薬物動態の検討試験の際は試験食品摂取前 12 時間から試験食品摂取後 3 時間までは絶食とする。

・飲料

被験者は試験食品摂取 24 時間前から試験食品摂取 48 時間後まで飲酒しない。試験食品摂取 24 時間前から試験食品摂取 48 時間後まで、飲料は水のみとする。

7.2. 有害事象

試験実施期間中はすべての有害事象 (自他覚症状や検査値異常など) について内容・発現時期・消失時期・程度・処置・転帰・重篤性評価を記録し、試験薬との関連性をカルテ、症例報告書(CRF) に記載する。必要に応じて追跡調査も行う。

7.3. 試験実施スケジュール

7.3.1. 研究スケジュール：絶食下における薬物動態の検討

	スクリーニング	study 1-3共通												
		試験食品摂取48時間前	24時間前	12時間前	1時間前	試験食品摂取	15分後	30分後	1時間後	2時間後	3時間後	6時間後	24時間後	48時間後
同意取得	○													
選択基準・除外基準	○													
併用薬	○				○								○	○
被験者背景	○													
自覚症状									○			○	○	○
摂取した食品の記録		←	―	―	―	―	―	―	―	―	―	―	―	→
食事：絶食				←	―	―	―	―	―	―	→			
食事：高ポリフェノールを避けた食事			←→									←	―	→
飲料制限（水のみ）			←	―	―	―	―	―	―	―	―	―	―	→
有害事象						←	―	―	―	―	―	―	―	→
身長・体重	○				○									○
バイタルサイン	○				○				○		○	○	○	○
診察	○				○				○			○	○	○
採血	○				○		○	○	○	○	○	○	○	○
血液一般検査	○				○									○
血中ロスマリン酸濃度					○		○	○	○	○	○	○	○	○

7.3.2. 研究スケジュール：食事摂取下における薬物動態の検討

	スクリーニング	study 4-6共通													
		試験食品摂取48時間前	24時間前	12時間前	1時間前	30分前食事摂取	試験食品摂取	15分後	30分後	1時間後	2時間後	3時間後	6時間後	24時間後	48時間後
同意取得	○														
選択基準・除外基準	○														
併用薬	○				○									○	○
被験者背景	○														
自覚症状										○			○	○	○
摂取した食品の記録		←	―	―	―	―	―	―	―	―	―	―	―	―	→
食事摂取*						●*									
高ポリフェノールを避けた食事			←	―	―	―	―	―	―	―	―	―	―	―	→
飲料制限（水のみ）			←	―	―	―	―	―	―	―	―	―	―	―	→
有害事象							←	―	―	―	―	―	―	―	→
身長・体重	○				○										○
バイタルサイン	○				○					○		○	○	○	○
診察	○				○					○			○	○	○
採血	○				○			○	○	○	○	○	○	○	○
血液一般検査	○				○										○
血中ロスマリン酸濃度					○			○	○	○	○	○	○	○	○

* 試験食品摂取 30-15 分前に被験者全員同じ食事（高ポリフェノールを避けた食事）を摂取する。

8. 試験の中止基準

8.1. 投与中止基準

研究期間中に下記の事例が発生した場合は投与を中止する。

(1). 重篤な有害事象の発現

(2). 同意の撤回

(3). その他研究責任医師による中止の必要性の判断

9. 有害事象の評価と報告

9.1 有害事象の定義

有害事象とは、試験錠剤が摂取された被験者に生じたあらゆる好ましくない医療上のできごとをいい、当該試験錠剤との因果関係の有無は問わないものである。

9.2 重篤な有害事象の定義

重篤な有害事象とは、試験錠剤が摂取された（摂取量にかかわらない）際に生じたあらゆる好ましくない医療上のできごとのうち、以下のものをいう。

a)死亡

b)死亡につながるおそれのある症例

c)入院または入院期間の延長が必要なもの

研究開始時から悪化していない既存の状態に対する処置のための入院は、有害事象とはみなさない。

d)障害／機能不全に陥る症例

9.3. 有害事象および重篤な有害事象の調査期間、頻度および方法

研究期間中の検査時に、担当医師または指名された者が被験者に有害事象に関する質問を行う。被験者の原資料には、同意文書に署名がなされた時から研究終了時まで、有害事象および重篤な有害事象が記録される。

9.4. 有害事象および重篤な有害事象の記録

担当医師は、有害事象／重篤な有害事象発現時に、当該事象に関連するすべての記録（例：病院での経過記録、臨床検査記録、診断報告書）を評価する責任を負う。担当医師は有害事象／重篤な有害事象に関する情報を症例報告書に記録する。担当医師は、徴候、症状／ほかの臨床上の情報に基づいて、その事象の診断名を特定するよう努める。この場合、個々の徴候／症状ではなく、診断名を有害事象／重篤な有害事象として記録する。

9.5. 研究終了後の有害事象と重篤な有害事象

研究終了後の有害事象／重篤な有害事象とは、本研究実施計画書の「7.3. 有害事象および重篤な有害事象の調査期間、頻度および方法」に定義された有害事象／重篤な有害事象の調査期間外に発現する事象のことである。担当医師は、研究終了後、被験者から有害事象または重篤な有害事象を積極的に収集する義務を負わない。ただし、被験者の研究終了後、死亡を含む重篤な有害事象を知り、試験食品と合理的な関連があるとみなされた場合は、担当医師はただちにこれを研究事務局に報告する。

9.6. 重篤な有害事象および予測できない新たな事象が発現した場合

試験責任医師または分担医師は適切な処置を行うとともに病院長・臨床研究審査委員会に速やかに報告する。

10. 評価項目

10.1. 主要評価項目(プライマリーエンドポイント)

主要評価項目として以下を含むレモンバーム由来ロスマリン酸製剤の薬物動態を評価する。

・レモンバーム由来ロスマリン酸製剤（100mg，250mg，500mg）服用後のロスマリン酸最高血中濃度（Cmax）、半減期($T_{1/2}$)、薬物血中濃度 - 時間曲線下面積(area under the curve: AUC)、絶食下および食事摂取下におけるロスマリン酸薬物動態の変化。

10.2. 副次的評価項目（セカンダリーエンドポイント）

副次的評価項目として以下を含む安全性および忍容性を評価する。

・有害事象発現頻度：研究期間の有害事象発現頻度を集計する。

・身体所見、バイタルサインおよび一般血液検査。

・ロスマリン酸 100mg, 250mg, 500mg 1回経口摂取の完遂率を算定する。

11. 統計的事項

11.1.薬物動態の解析

11.1.1. 主要評価項目

11.1.1.1. 薬物動態

絶食下および食事摂取下の試験それぞれについてレモンバーム由来ロスマリン酸製剤（100mg/250mg/500mg）服用後のロスマリン酸の Cmax、$T_{1/2}$ はロスマリン酸服用者(絶食下試験、食事摂取下試験各 n=9)について平均値±標準誤差を算出する。AUC は台形公式（trapezolital rule）を用いて算出し、ロスマリン酸服用者（絶食下試験、食事摂取下試験各 n=9）について平均値±標準誤差を算出する。

絶食と食事摂取下のいずれが薬物の吸収に優れているか検討する。

11.1.2. 副次的評価項目

11.1.2.1. 忍容性

ロスマリン酸（100mg/250mg/500mg）1回経口摂取の忍容性を算出する。本登録された被験者数を分母とし、ロスマリン酸に割りつけられた被験者が試験食品を内服できた数を分子とする。

11.1.2.2. 有害事象

有害事象は有害事象共通用語基準をもちいてコード化する。事象の頻度、被験者の頻度、および各有害事象を報告した被験者の割合は全有害事象および錠剤に関連する有害事象について個別に摂取群毎にまとめる。少なくとも1件の有害事象、少なくとも1件のロスマリン酸に関連する有害事象、少なくとも1件の重篤な有害事象、および少なくとも1件の脱落を引き起こす有害事象を報告した被験者の割合を摂取群毎に算出する。Fisher の直接確率法を用いて、この一連の各有害事象についての摂取群間の比較をおこなう。

11.2. 解析対象被験者の規定

有効性および安全性の統計解析に使用する主要な被験者集団は、試験錠剤群に無作為に割り付けられ、試験錠剤を少なくとも1回服用した被験者から構成される FAS(full analysis set)集団とする。PPS(per protocol set)集団は FAS 集団から重大な研究実施計画書違反があった患者を除いた被験者から構成される。重大な研究実施計画書違反には、選択除外基準からの逸脱、累積摂取遵守率＜75%、間違った試験錠剤の服用が含まれる。FAS 集団の被験者の 20%以上が重大な研究実施計画書違反をひとつまたは複数経験していた場合には、PPS 集団を用いて主要な有効性の解析をおこなう。

12. 症例報告書の記入と報告

下記の事項を記載する。

1. 作成・修正・追記の方法

・黒色のボールペンで記入する。

・症例報告書の記入内容を変更または修正する場合には×または二重線にて修正する。変更または修正（追記、削除も含む）箇所には年月日を記入し研究責任医師（分担医師）が捺印または署名する。

・観察・検査項目に規定する項目の観察・検査項目結果は診療録に記載すること。本研究で定める症例報告書にそれらの結果を転記すること。

2. 中止もしくは逸脱被験者の取り扱い

・中止もしくは逸脱被験者においても可能な限り追跡調査を行い症例報告書に記載する。

13. 倫理的配慮

本研究の実施にあたってはGCP、ヘルシンキ宣言、臨床研究に関する倫理指針に準拠する。

13.1. インフォームド・コンセントのための手続き及び方法

被験者には検査前に研究計画を説明し、インフォームド・コンセントを得て、別紙の説明書および同意書に署名してもらう。

13.1.1. 説明書

下記項目を同意説明文中に記載し、臨床研究の概要がつかめるものとする。
① 臨床研究の目的。
② 臨床研究の方法。
③ 被験者の臨床研究への参加予定期間。
④ 臨床研究に参加する予定の被験者数。
⑤ 予期される臨床上の利益および危険性または不便。
⑥ 当該疾患に対する他の治療方法の有無およびその治療方法に関して予期される重要な利益および危険性。
⑦ 臨床研究への参加は被験者の自由意思によるもので、臨床研究への参加を随時拒否、撤回できること。また、これによって被験者が不利な扱いを受けないこと。
⑧ 臨床研究への参加を中止させる場合の条件または理由。
⑨ 臨床研究の結果が公表される場合であっても被験者の秘密は保全されること。
⑩ 臨床研究に参加した場合の費用と謝金、健康被害を受けた場合の治療および補償。
⑪ 他の薬剤に関する事項。
⑫ 臨床研究責任医師の氏名・職名・連絡先。
⑬ 被験者が守るべき事項。
⑭ 当該臨床研究に関する照会連絡先。(18. 研究組織参照)

13.1. 2. 同意書

説明書の内容を理解し、神経疾患に関する臨床研究に参加することについての同意の有無を確認できるものとする。金沢大学附属病院様式に従うことを原則とし、必ず文書にて取得する。

13.2. 起こりうる危険や不利益などについて

以前に海外でおこなわれたレモンバームから抽出したロスマリン酸約240mg/日16週間の投与プラセボ対照二重盲検比較試験において、嘔吐、浮動性めまい、喘鳴、興奮、腹痛、吐き気が有害事象として報告されたがいずれもプラセボ群に比して有意差はなかった[4)]。レモンバームには弱い鎮静作用があるとされているため[7)]、レモンバーム抽出ロスマリン酸投与により傾眠傾向が生じるおそれがある。

採血は看護師または医師により行われ、危険性はほとんどない。

13.3. 健康被害が生じた場合の補償・賠償

・本研究に起因して、症例に何らかの健康被害が発生した場合は、医療機関は治療その他必要な措置を講ずるものとし、治療に要した医療費の内健康保険等からの給付を除く症例の自己負担額は各症例が負担する。

13.4. 個人情報の保護の方法

13.4.1. 資料等の保存方法

個人情報や検査で得られたデータは、連結可能匿名化し対応表は金沢大学神経内科で厳重に管理する。個人情報の保護に関しては、紛失を防ぐため、対応表を電子データと紙の文書の両方で管理する。電子データはパスワードが必要なコンピューターに入れてパスワードは個人情報管理責任者

のみが知っている。コンピューターはインターネットには接続しない。紙の文書は個人情報管理責任者のもと、施錠できる研究室の金庫に保管し、その鍵は個人情報管理者が管理する。学会などで研究結果を公表する際には個人が特定できないように配慮し匿名性を守る。

個人情報分担管理者：岩佐　和夫（医薬保健研究域医学系准教授）

13.4.2. 研究終了後の資料等の取り扱い

研究上用いた個人情報（電子媒体によるものを含む）等は、以後研究に支障のないものについては細段等の復元不可能な方法により適切に処理する。

採血検査施行後の残存検体については、試験食品について未知の合併症が生じた場合等に血液、マーカーの経時的変化を評価したり、別の医学研究に使用するために、保存の同意を得て金沢大学医薬保健研究域医学系（脳老化・神経病態学）にて保存する。将来別の医学研究に使用する場合には再度倫理委員会等適切な倫理審査委員会の承認を得たのちに検体を使用する。

14. 費用について

この試験に対する、特定の企業からの資金提供はない。神経内科学講座の委任経理金あるいは本研究のために確保する研究費を用いて実施する。

試験食品（レモンバーム抽出ロスマリン酸カプセル及びプラセボカプセル）については丸善製薬株式会社より無償提供をうける。

15. 実施計画の変更について

研究中に本実施計画を変更する場合は、金沢大学医学倫理委員会に申告、承認を得たのちに計画を変更する。

16. 記録の保存について

①保存すべき資料は診療録、ロスマリン酸血中濃度検査票、症例報告書（CRF）である。
②保存期間および保存場所、保存責任者
保存期間は 5 年間、保存場所は神経内科学講座、保存責任者は研究責任医師とする。

17. 研究成果の帰属と結果の公表

研究成果から生じる知的財産権は金沢大学医薬保健研究域医学系　脳老化・神経病態学（神経内科）に帰属する。解析を行い学会発表および学術雑誌に掲載する。ただし、個人の特定が可能となるような属性については秘匿措置を講ずる。

18. 研究組織

研究責任者および分担者

(1). 研究責任者
金沢大学大学院　脳老化・神経病態学分野　教授　山田正仁
〒920-0934 石川県金沢市宝町 13-1, TEL 076-265-2000

(2). 研究分担者

氏名	所属	職名
岩佐　和夫	金沢大学医薬保健研究域医学系	准教授
小野　賢二郎	金沢大学附属病院	助教
篠原　もえ子	金沢大学附属病院	医員
小林　彰子	東京大学大学院農学生命科学研究科	准教授
永井　俊匡	高崎健康福祉大学健康福祉学部	講師

19. 参考文献

1) Petersen M, Simmonds MS: Rosmarinic acid. Phytochemistry 2003, 62: 121-125.
2) Ono K, Yoshiike Y, Takashima A, et al. Potent anti-amyloidogenic and fibril-destabilizing effects of polyphenols in vitro: implications for the prevention and therapeutics of Alzheimer's disease. J Neurochem 2003, 87: 172-181.
3) Hamaguchi T, Ono K, Murase A, et al. Phenoic compounds prevent Alzheimer's pathology through different effects on the amyloid-β aggregation pathway. Am J Pathol 2009, 175: 2557-2565.
4) Akhondzadeh S, Norozian M, Mohammadi M, et al. Melissa officinalis extract in the treatment of patients with mild to moderate Alzheimer's disease: a double blind, randomized, placebo controlled trial. J Neurol Neurosurg Psychiatry 2003, 74: 863-866.
5) Merrill RA. Regulatory toxicology. In: Klaassen CD (ed) Toxicology, the basic science of poisons, 5th edn. McGraw-Hill, New-York, 1996, 1011-1023.
6) Natural medicines comprehensive database (available at: http://www.naturaldatabase.com/)
7) Kennedy DO, Scholey AB, Tildesley NT, et al. Modulation of mood and cognitive performamce following acute administration of Melissa officinalis (lemon balm). Pharmacol Biochem Behav 2002; 72: 953-964.
8) Kennedy DO, Wake G, Savelev S,et al. Modulation of mood and cognitive performance following acute administration of single doses of Melissa Officinalis (Lemon Balm) with human CNS Nicotinic and Muscarinic Receptor-Binding Properties. Neuropsychopharmacology 2003; 28: 1871-1881.
9) Baba S, Osakabe N, Natsume M, et al. Absorption, metabolism, degradation and urinary excretion of rosmarinic acid after intake of Perilla frutescens extract in humans. Eur J Nutr 2005, 44: 1-9.
